# Liquid Gold with a Dark Side—A Toxicological Overview of Bioactive Components in Honey

**DOI:** 10.3390/molecules30193925

**Published:** 2025-09-29

**Authors:** Maciej Kulawik, Anna Kulawik, Judyta Cielecka-Piontek, Przemysław Zalewski

**Affiliations:** 1 Department of Pharmacognosy and Biomaterials, Poznan University of Medical Sciences, Rokietnicka 3, 60-806 Poznan, Poland; maciej.kulawik@student.ump.edu.pl (M.K.); anna.kulawik@student.ump.edu.pl (A.K.); jpiontek@ump.edu.pl (J.C.-P.); 2 Doctoral School, Poznan University of Medical Sciences, Bukowska 70 St., 60-812 Poznan, Poland

**Keywords:** mad honey, pyrrolizidine alkaloids, grayanotoxins, triptolide, celastrol, gelseine-type alkaloids, tropane alkaloids, tutin, coumarins, 5-hydroxymethylfurfural

## Abstract

Honey is a valuable natural product prized for its nutritional and therapeutic properties, including antioxidant, antimicrobial, and anti-inflammatory effects. However, in addition to health-promoting compounds, honey may also contain plant-derived toxins, contaminants, and degradation products. Certain phytotoxins—such as pyrrolizidine alkaloids, grayanotoxins, triptolide, celastrol, gelsedine-type alkaloids, and tutin—can be transferred to honey from specific plant sources and pose health risks, particularly at high doses or with long-term exposure. Furthermore, compounds like 5-hydroxymethylfurfural, trace metals, pesticide residues, and *Clostridium botulinum* spores may present additional risks, especially to sensitive groups such as infants. Consumers often assume that natural products are inherently safe, which may lead to unintentional exposure to harmful substances. Adverse effects can range from chronic toxicity to, in extreme cases, death. Therefore, raising awareness among consumers and vendors is essential to reduce the intake of honey from unverified sources. Continuous monitoring of honey composition and further studies on the toxicodynamics of rare contaminants are crucial to ensuring safety while preserving the therapeutic benefits of this remarkable natural substance.

## 1. Honey

Honey is a natural product made by bees using floral nectar or honeydew [1]. Approximately 300 distinct types of honey have been identified, with their diversity primarily determined by the floral nectar sources and the climatic and environmental conditions in the production regions [2]. It may be classified as floral when sourced from nectar, non-floral when derived from plant secretions, or mixed if it results from a combination of both [3]. Nectar honey is divided based on its botanical origin into monofloral, when derived predominantly from the nectar or honeydew of a single plant species, and multifloral, when it originates from multiple botanical sources [4]. Honeydew honey is primarily derived from the sugary secretions of plant-sucking insects (*Hemiptera*) feeding on the living parts of plants, or directly from plant exudates [5].

Upon returning to the hive, nectar-collecting honey bees promptly transfer their nectar to food-storer bees through trophallactic exchange. These recipient bees then deposit the nectar into various comb cells for further processing [6]. The transformation of floral nectar into honey begins with a reduction in water content. Foragers actively promote evaporation by regurgitating the nectar and holding it between their mandibles to facilitate water loss [7]. Additionally, nectar undergoes passive evaporation within the cells of the hive, and studies indicate that its repeated relocation among cells before final deposition plays a critical role in the maturation of honey [8]. Notably, water removal may also initiate during the return flight to the hive [9]. Concurrently, sucrose in the nectar is enzymatically hydrolyzed by invertase, which is secreted into the nectar while it resides in the bee’s crop during collection [6]. Full maturation of honey typically requires around thirty-six days, although the exact duration depends on environmental factors such as ambient temperature, humidity, and the initial content of the water in the nectar [10].

Honey is widely recognized as a natural, nutritious, and health-promoting food product, whose chemical composition is subject to considerable variation depending on its botanical origin and geographical location [11]. Natural honey is composed predominantly of carbohydrates (approximately 80–85%), primarily glucose and fructose, along with 15–17% water, 0.1–0.4% proteins, and about 0.2% ash [12]. It also contains small amounts of amino acids, vitamins (i.e., vitamin C, thiamine, niacin, riboflavin, pantothenic acid, and vitamin B6), enzymes (i.e., catalase, glucose oxidase, and superoxide dismutase), and other bioactive compounds, including phenolic antioxidants [12,13]. The color of honey varies from water white to dark amber and is primarily influenced by its content of phenolic compounds and minerals [14,15].

Honey represents a premium food product that is widely appreciated for its distinctive flavor, nutritional value, and health-promoting properties, all of which are closely linked to its chemical composition. Its production and commercialization occur worldwide, with notable variations in nutritional content and biological activity [16]. Honey’s medicinal properties can be utilized through oral intake or external application, depending on the intended therapeutic outcome [17].

The therapeutic potential of honey has been recognized in medical practice since antiquity [18]. Many current in vitro and in vivo studies indicate a broad spectrum of honey activity, including antioxidant, anti-inflammatory, antiviral, antibacterial, antidiabetic, and anticancer properties [17,19]. Its antioxidant potential is attributed to the presence of phenolic compounds and flavonoids, which help neutralize free radicals and mitigate oxidative stress [20]. Research also indicates its preventive effect on diseases of the nervous, respiratory, cardiovascular, and digestive systems [19]. The topical use of honey is indicated for a range of conditions, including inflammatory skin disorders (e.g., eczema), viral lesions, acute and chronic wounds, thermal injuries, surgical wound healing, and fungal infections [21].

One of the most common traditional uses of honey is in the treatment of respiratory tract infections. It has been shown to be a safe and potentially effective option for relieving cough associated with upper respiratory infections, particularly in children [22]. Clinical studies indicate that it may reduce cough frequency and severity better than no treatment, placebo, or diphenhydramine, and in some cases, even salbutamol [23,24]. A small bedtime dose can alleviate night-time cough and improve sleep quality in both children and parents [25]. Its therapeutic action may be linked to its antimicrobial and anti-inflammatory properties [23,26].

Honey is considered a valuable dietary product for humans due to its high nutritional value (304 kcal per 100 g) and its content of readily absorbable carbohydrates [27]. It has viscosity 36.1 mPa·s at 25 °C [28]. The moisture content of honey is strictly regulated by quality standards. In most types of honey, it should not exceed 20%, while for heather honey (*Calluna*), the permissible limit is 23% [29]. The viscosity of honey depends mainly on its sugar and water content—high glucose content and low moisture increase viscosity, which affects crystallization, sensory properties, and product stability [30]. Honey has a density of about 1360 kg/m^3^. This makes it 40% denser than water. Additionally, honey exhibits an acidic pH, typically ranging from 3.2 to 4.5, along with high osmotic pressure and low water content, all of which contribute to its physicochemical stability [27,31].

The assessment of honey authenticity and quality represents a crucial area of applied research, with substantial implications for the food industry. The concept of honey authenticity is defined by the Codex Alimentarius, the EU Honey Directive, and various national regulations [4,29]. However, while these regulations provide a framework for defining honey authenticity, they offer limited guidance on addressing the presence of undesirable substances that may compromise honey quality. This review aims to highlight this regulatory gap and support ongoing efforts to enhance consumer protection and product integrity.

Honey produced in EU countries or marketed within EU territory must comply with specific compositional standards regarding carbohydrate content, acidity, moisture level, diastase activity, electrical conductivity, and 5-hydroxymethylfurfural (HMF) concentration, in order to ensure its authenticity, safety, and quality [32]. The composition of honey—particularly its high fructose content, presence of organic acids, and low pH—promotes the formation of HMF, whose concentration increases with heat treatment and prolonged storage [33].

Monitoring and evaluating the quality of honey is essential, as it may be subject to various forms of contamination. Honey may become contaminated due to environmental pollutants, such as heavy metals, polychlorinated biphenyls, and pesticides originating from treated or rotational crops. Contamination may also result from inappropriate beekeeping practices, including the use of veterinary drugs and insecticides within the hive, as well as from naturally occurring plant toxins [34].

In the detection of contaminants in honey, a wide range of analytical methods is used, allowing for the identification of pesticide residues, antibiotics, microorganisms, as well as heavy metals. Pesticides are most commonly determined using chromatographic techniques, such as gas chromatography coupled with mass spectrometry (GC-MS, GC-MS/MS) and liquid chromatography (LC-MS, UHPLC-MS/MS). Sample extraction and cleanup techniques are also employed, e.g., liquid–liquid extraction (LLE), solid-phase extraction (SPE), or its variant using magnetic nanoparticles (MSPE), which allow selective isolation of compounds from the honey matrix. Antibiotics are detected using screening methods and confirmatory methods based on chromatography coupled with mass spectrometry (LC-MS/MS). Microorganisms in honey are identified using classical microbiological methods (cultures on media, biochemical tests, staining) as well as modern techniques: PCR and its variants, 16S rRNA sequencing or the proteomic MALDI-TOF MS technique. Heavy metals and metalloids are determined using spectroscopic methods such as atomic absorption spectroscopy (AAS), inductively coupled plasma atomic emission spectroscopy (ICP-AES, ICP-OES), or X-ray fluorescence (TXRF) [34].

Honey is widely consumed worldwide, and while its beneficial properties are well known, it can also contain toxic compounds derived from plants, environmental contaminants, or residues of agrochemicals. Although some of these substances are rare or region-specific, their potential for acute or long-term toxicity makes them relevant from a food safety perspective. This review focuses primarily on honeys produced by *Apis mellifera* and aims to provide a comprehensive overview of both common and less frequently studied toxic and bioactive components in honey, highlighting their sources, mechanisms of toxicity, and potential health risks. By summarizing current knowledge and identifying gaps in the literature, this work contributes to a better understanding of honey safety and informs future research and regulatory considerations.

## 2. Mechanism of Toxin Transfer to Honey

Honey contamination can occur through multiple environmental and anthropogenic pathways. Flowers, the primary source of nectar and pollen for bees, may contain residues of harmful substances such as heavy metals and pesticides, deposited from polluted air, soil, or water [35,36]. Bees act as vectors and can introduce these contaminants into the hive both internally, by collecting contaminated nectar and pollen, and externally, by carrying particles on their bodies. As honey matures, undergoing enzymatic transformation of sucrose and a gradual reduction in water content, the concentration of these impurities may increase. One of the most significant sources of contamination is the presence of pesticide residues. Bees may unknowingly forage on plants treated with agrochemicals, thereby transferring harmful compounds into the honey they produce. In addition, heavy metals can contaminate honey due to environmental exposure. Their presence is often associated with industrial activity or agricultural runoff and reflects the overall level of environmental pollution in a given area [34,37,38]. Another relevant source of contamination is the use of veterinary medicinal products. Beekeepers sometimes apply antibiotics to control diseases in bee colonies, whether legally or illegally, which can lead to the presence of antibiotic residues in honey [39,40]. Not all beekeepers comply with legal regulations, and some may administer veterinary drugs in excessive doses without veterinary supervision, further increasing the risk of contamination.

In contrast to pollutants, honey can also be contaminated with naturally occurring plant toxins. A notable example is pyrrolizidine alkaloids (PAs), introduced when bees forage on plants that biosynthesize these compounds [41,42]. Bees can introduce these alkaloids into the hive when collecting nectar or pollen from such plants. These particles can subsequently enter the hive and become incorporated into honey. In addition, airborne pollen grains from PAs-producing plants may enter the beehive through natural airflow and contribute to the contamination of stored honey [43].

The final concentration of contaminants in honey is not uniform but varies depending on multiple ecological and geographical factors, including landscape type, season, floral source, and local biodiversity.

Contaminants in honey affect its quality and marketability [44,45]. They also pose risks to consumer health and compromise its therapeutic and nutritional properties. Addressing this challenge requires the implementation of stringent regulations throughout the entire production chain, alongside robust quality control systems. This involves continuous monitoring of environmental pollutants, compliance with permissible limits for pesticide and antibiotic residues, and comprehensive safety testing. Moreover, it is essential to mitigate contamination at its source by promoting sustainable agricultural practices and responsible use of antibiotics in apiculture [46].

## 3. Phytotoxins in Honey

### 3.1. Pyrrolizidine Alkaloids

Pyrrolizidine alkaloids (PAs) are widespread plant metabolites found in many species. They are most commonly present in the genera *Senecio* (*Asteraceae*), *Echium* (*Boraginaceae*), *Crotalaria* (*Fabaceae*), and *Eupatorium* (*Asteraceae*) [47,48]. They usually occur as esters—monoesters, diesters, or macrocyclic diesters—composed of a necine base (amino alcohols) and necic acids (mono- or dicarboxylic aliphatic acids), which accounts for their structural diversity [49]. PAs rarely occur in free form; they are most commonly present as tertiary bases or N-oxides (PANOs) [50]. The pyrrolizidine core consists of two fused five-membered rings with a nitrogen atom, and sometimes a double bond at the 1,2-position, which increases toxicity. Necine bases may carry hydroxyl groups at positions C-1, C-2, C-6, or C-7, while esterification typically occurs at C-7 and/or C-9 [49].

**Figure 1:**
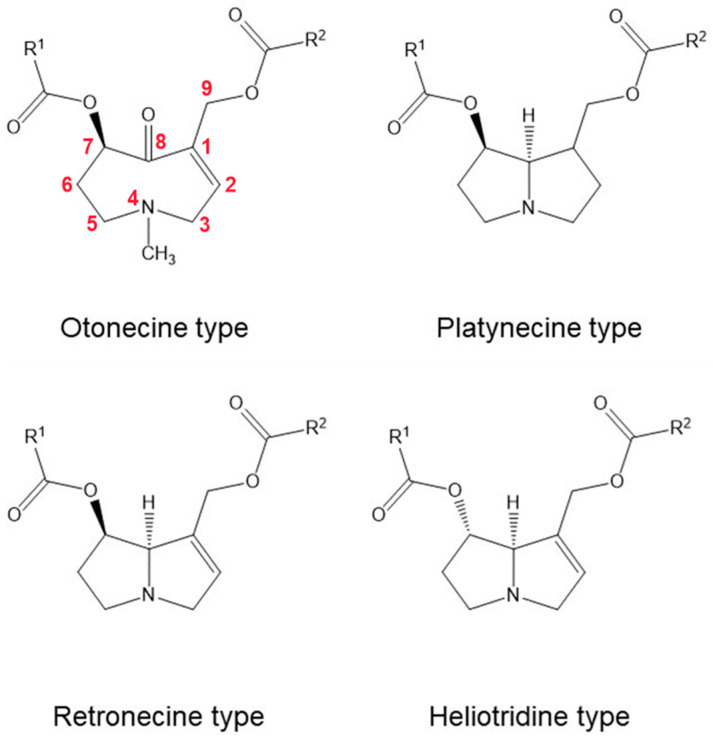
Chemical Structures of Selected PAs: Otonecine, Platynecine, Retronecine, Heliotridine types.

PAs (Figure 1) are classified into four groups based on the structure of the necine base: retronecine, heliotridine, otonecine (with a monocyclic ring oxidized at C-8), and platynecine (saturated structure). Retronecine and heliotridine are diastereoisomers differing in their stereochemistry at C-7. Necic acids are aliphatic carboxylic acids such as angelic, tiglic, trachelanthic, viridifloric, senecic, or isatine acid. The combination of necine bases and necic acids results in monoesters or diesters, including those characteristic of the *Boraginaceae* family—esters with a hydroxyl group at C-9—or macrocyclic diesters typical of the *Asteraceae*, in which both C-7 and C-9 are esterified with a dicarboxylic acid. Less commonly, necine bases are esterified with aromatic or arylalkyl acids [49].

PAs are recognized as potentially carcinogenic compounds [51,52]. Moreover, they exhibit toxicity and genotoxicity which are associated with their metabolic activation pathways in the body. PAs are metabolized in the liver by cytochrome P450 enzymes into reactive metabolites. These bioactive compounds can damage hepatocytes, leading to necrosis, hepatic steatosis, and diseases such as hepatic veno-occlusive disease. The reactive metabolites bind to DNA, forming exogenous DNA adducts. These adducts can cause DNA damage, such as mutations, which are critical in the initiation of carcinogenic processes [53]. It should be emphasized that there is a lack of research on the long-term effects of PAs consumption. Current studies focus mainly on the acute toxicity of selected compounds and may not be representative of effects in humans. To date, however, there is a lack of epidemiological evidence in humans confirming the carcinogenicity of PAs.

PAs are present in plants mainly as N-oxides, while in honeys they occur predominantly in the free base form. It is assumed that the reduction in PANOs may take place in the digestive system of bees [47]. In a study conducted on Italian multi- and monofloral honeys, PAs concentrations ranged from 0.9 to 33.11 μg/kg [54]. In the study of Polish honey samples, PAs were detected in 16 samples (32%), with echimidine, lycopsamine, and intermedine being the most frequently occurring. PAs concentrations ranged from 1,4 to 52.4 µg/kg [42]. In another study analyzing 32 honey samples from various countries, PAs were detected in 60% of them, with total concentrations ranging from 1.4 to 14.2 µg/kg for 12 reference compounds. After applying a semi-quantitative model, it was found that the total concentration of PAs in some samples could reach as high as 117 µg/kg [55]. PAs are widely distributed in food—they are found in cereals, teas, herbal products, spices, and dietary supplements, and honey is not their only source in the human diet [56].

### 3.2. Grayanotoxins

Grayanotoxins (GTXs) are toxic compounds derived from the leaves, twigs, or flowers of plants in the *Ericaceae* family, such as *Rhododendron*, *Pieris*, *Agarista*, and *Kalmia* [57]. Plants containing GTXs are found in the eastern regions of the Black Sea area in Turkey, as well as in South America, Europe, and Japan. Cases have also been reported in Spain, Portugal, Brazil, the United States, and Nepal [58]. Chemical structures of GTXs are those of a diterpene—a cyclic, polyhydroxylated carbohydrate with a 5/7/6/5 ring system and no nitrogen. More than 25 isoforms of GTXs have been identified [59]. GTXs are soluble in both water and lipids. In *Rhododendron* species, grayanotoxin III (GTX-III) is the predominant compound and is considered highly toxic. Grayanotoxin I (GTX-I), which is also highly toxic, as well as grayanotoxin II (GTX-II), are usually present in smaller [57].

**Figure 2:**
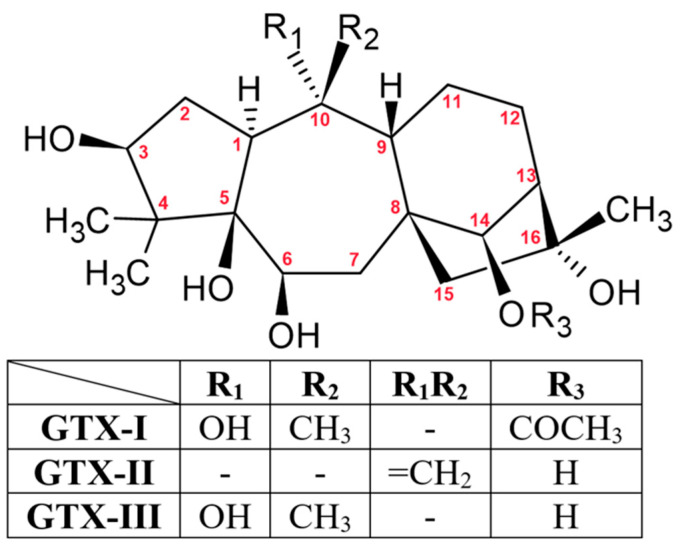
Chemical Structures of GTXs I-III.

GTXs (Figure 2) act on sodium channels in cells, primarily in muscle and nerve tissue. They bind to open channels, preventing their inactivation. This increases membrane permeability, prolongs depolarization, and inhibits repolarization. These effects resemble cholinergic activity, leading to bradycardia, hypotension, and respiratory depression. The toxins also cause dysfunction of the sinoatrial node by reducing the action potential. Sodium channels in skeletal muscles are more sensitive to GTXs than those in cardiac muscle. GTXs also show affinity for M2 muscarinic receptors, contributing to bradycardia and cardiac toxicity [60]. Moreover, GTXs may be toxic to the testes, especially at high doses and with chronic exposure [57].

A study conducted on rats demonstrated that honey containing GTXs induced oxidative stress and genotoxicity [61]. The high content of GTXs in the nectar of *Rhododendron ponticum* and *Rhododendron luteum* poses a challenge for beekeepers in regions where these species naturally occur. Honey produced by bees in these areas may contain significant amounts of toxins [62]. Honey contaminated with GTXs is referred to as “mad honey” due to the symptoms it induces. A small amount of mad honey is sufficient to cause toxicity. The average ingested dose ranged from 5 to 30 g. Symptoms appeared within 30 min to 3 h after consumption. Many sources indicate that this type of honey has a characteristic, intense, and burning taste [63]. Turkey is a country where poisonings occur frequently due to the local flora [64]. In honeys originating from Nepal, the content of GTX-I ranged from 0.75 to 64.86 μg/g, while GTX-III ranged from 0.25 to 63.99 μg/g [65]. There is little data on the level of GTX-II due to its relatively lower toxicity.

In alternative medicine, mad honey is used for a variety of conditions, including high blood pressure, diabetes, influenza, digestive issues, arthritis, various viral infections, skin disorders, general pain, and the common cold [64]. It is often promoted as a remedy for sexual dysfunction. The group more frequently affected by poisoning consists of older men [66]. This correlation may result from age-related declines in potency and attempts to supplement it using traditional or folk methods [67]. The toxin content does not decrease during storage; therefore, mad honey is not deactivated over time while being stored [62]. The European Food Safety Authority (EFSA) proposes that the maximum concentration for the sum of GTX-I and GTX-III in honey should be 0.05 mg/kg of honey [68]. However, it is important to note that this value applies only to these two toxins and does not take into account other compounds present in honey.

### 3.3. Triptolide

There is inconsistency in the literature regarding the term “mad honey”. In addition to honey contaminated with GTXs, this term may also refer to products containing triptolide [69].

In East Asia, cases have been reported of honey containing a toxin derived from plants of the *Tripterygium* genus (family *Celastraceae*), which primarily grow in the mountainous regions of East Asia [70].

**Figure 3:**
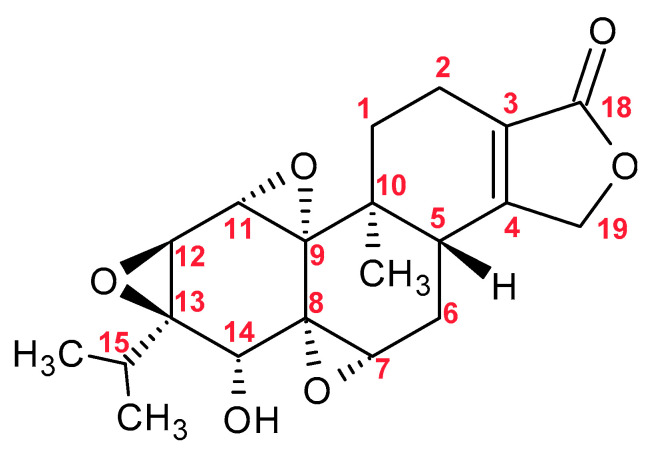
Chemical Structure of Triptolide.

Triptolide (Figure 3) is a bioactive diterpenoid compound with strong anti-inflammatory, immunosuppressive, and anticancer properties. However, its high toxicity significantly limits its therapeutic applications. It causes damage to multiple organs, including the liver, kidneys, heart, and reproductive system. The mechanism of its toxicity involves oxidative stress, mitochondrial damage, apoptosis, cell cycle disruption, and hormonal imbalances [71].

The presence of triptolide in honey has been linked to acute poisonings that can lead to multiple organ failure and even death. In southwestern China, a series of poisonings occurred following the consumption of wild honey, affecting three previously healthy men. Symptoms appeared within 19–48 h and included severe nausea, vomiting, diarrhea, fever, dizziness, and intense back pain. One patient developed anuria and skin petechiae, and his condition rapidly deteriorated, resulting in death due to acute kidney failure and toxic myocarditis. The other two patients survived, although they continued to experience back pain even after two weeks of treatment. The poisoning was clustered and associated with the consumption of dark-colored honey with a bitter taste. Notably, seven other individuals who drank boiled water mixed with the same honey showed no symptoms [70].

In a study conducted by Sun et al., honey and flower samples of *Tripterygium wilfordii* (TwHf) from six apiaries in China were analyzed. Triptolide was detected in all honey and flower samples originating from TwHf, with concentrations in honey ranging from 396 to 633 µg/kg. This compound was not found in any of the 61 samples of typical honey or in the 90 commercial samples available on the market. As a result, triptolide was identified as a unique marker that allows for the unambiguous identification of “mad honey” derived from *Tripterygium wilfordii* [69].

In a study conducted by Xiao et al., the presence of toxic chemical compounds derived from TwHf in raw honey was investigated, with particular focus on triptolide. The authors identified five compounds in TwHf flowers, with triptolide present at the highest concentration (1.52 mg/kg). To assess triptolide’s toxicity, acute toxicity tests were performed on zebrafish larvae (*Danio rerio*), examining its effects in pure form as well as in combination with honey, artificial honey (mimic honey), and simple sugars (glucose and fructose). Triptolide exhibited strong toxic activity (LC_50_ = 0.28 µg/mL), which significantly increased when mixed with honey (LC_50_ = 0.11 µg/mL) and its components. A synergistic effect was observed, with its intensity decreasing in the following order: honey/artificial honey > glucose > fructose. Histopathological analysis revealed that the main target organs of triptolide toxicity were the heart and liver—manifested by pericardial edema, hepatocyte necrosis, and delayed yolk sac absorption [72].

Triptolide is a compound with proven toxicity to humans, and cases of poisoning caused by consumption of honey containing this substance have been reported in the literature. On the other hand, this compound is a poorly characterized toxin that may occur in honey. Animal studies indicate its high toxicity, which is consistent with the reported cases of poisoning.

### 3.4. Celastrol

**Figure 4:**
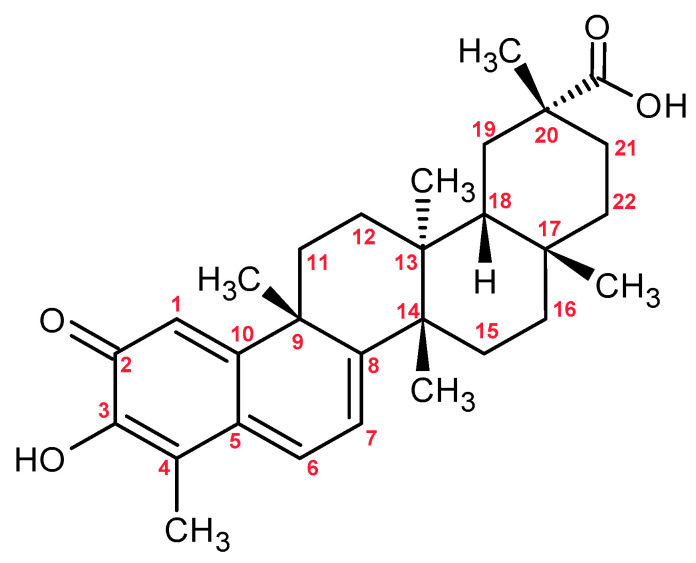
Chemical Structure of Celastrol.

Celastrol (Figure 4) is a natural compound belonging to the class of pentacyclic nor-triterpenoids, with a structure based on the oleanane skeleton. Its molecular framework includes characteristic functional groups such as a hydroxyl group at the C-3 position, a ketone group (=O) at C-6, and a carboxyl group (-COOH) at C-29. Additionally, it contains a quinone methide system, which is responsible for its high biological reactivity, including the ability to form Michael adducts [73]. This compound exhibits strong anticancer, anti-inflammatory, and antioxidant properties. It acts by inducing apoptosis, causing cell cycle arrest, inhibiting angiogenesis and metastasis, and modulating multiple signaling pathways (e.g., NF-κB, Akt/mTOR, JAK2/STAT3) [74]. Despite its high therapeutic potential, its application is limited by low bioavailability, a narrow therapeutic window, and adverse effects [74,75]. Studies have shown that celastrol may exhibit cytotoxic, hepatotoxic, and even neurotoxic effects at micromolar concentrations or with prolonged exposure. For example, exposure of primary rat hepatocytes to 3 µM celastrol caused significant cell damage, while zebrafish embryos displayed pericardial edema and malformations at 0.5–1.5 µM, with complete lethality at 2 µM. In animal models, doses as low as 0.5–2 mg/kg induced cardiac and hepatic injury, and administration of 5 mg/kg led to marked hematopoietic toxicity [76,77].

Xiao et al. identified celastrol as one of the main toxins derived from TwHf. Although its concentration in TwHf flowers was relatively low (17.43 µg/kg), it was the only compound detected in one of the raw honey samples from a high-risk area (Hubei), where its content reached 42.33 ng/kg. In acute toxicity tests on zebrafish larvae, celastrol exhibited strong toxic effects (LC_50_ = 0.18 µg/mL), which were significantly enhanced in the presence of honey (LC_50_ = 0.06 µg/mL), artificial honey (LC_50_ = 0.03 µg/mL), and sugars, especially glucose. Histopathological analysis showed that celastrol caused damage to the heart and liver, including pericardial edema, a reduced number of cardiomyocytes, and hepatocyte necrosis. These findings suggest that celastrol may be one of the key factors responsible for the toxicity of so-called “poisonous honey” derived from TwHf [72].

Due to the lack of studies describing the effects of consuming honey containing celastrol, further research is needed on this compound and its impact on pollinators. Given the reported cases of poisoning from TwHf-derived honey, it is not possible to clearly determine whether the toxicity was caused by triptolide, celastrol, or their synergistic action. This should be taken into account when conducting more detailed studies. Despite the high toxicity of this compound in animal models, the amount of celastrol in honey is very low, which significantly reduces the risk of poisoning after a single consumption.

### 3.5. Gelsedine-Type Alkaloids

Gelsedine-type alkaloids are natural compounds found in plants of the *Gelsemium* genus, which grow primarily in subtropical and tropical regions of Asia and North America [78,79]. These plants are known for their toxicity, but have been used for centuries in traditional Asian medicine, e.g., to treat skin ulcers, inflammations, and other ailments. Gelsedine-type alkaloids are characterized by a complex, polycyclic structure that includes: a common oxabicyclo[3.2.2]nonane core, a spiro-N-methoxyindolinone moiety, and various heterocyclic groups (such as pyrrolidine, pyrroline, pyrrolidinone, or azetidine), embedded within compact, three-dimensionally intricate molecular skeletons [79]. An example of a compound belonging to the gelsedine-type alkaloids is gelsenicine. It exhibits neurotoxic effects, causing respiratory depression and seizures [80].

**Figure 5:**
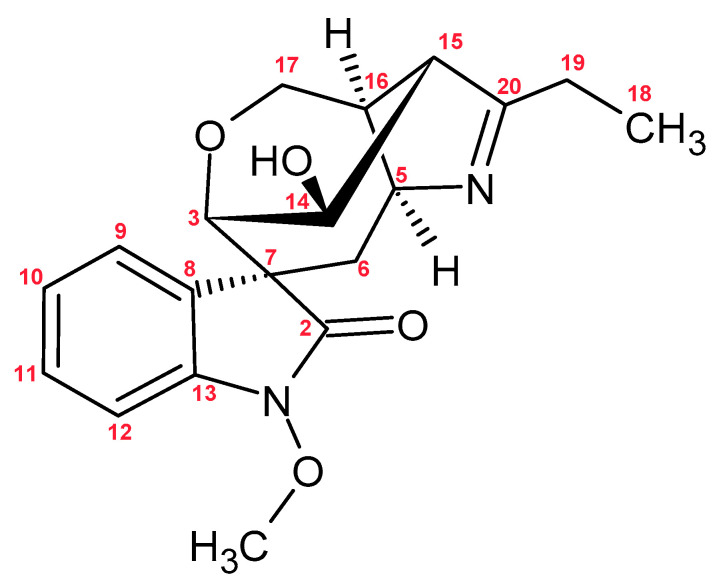
Chemical structure of 14-(R)-hydroxy-gelsenicine.

In a study conducted by Yang et al., the detection and detailed characterization of a new natural neurotoxin in honey—14-(R)-hydroxy-gelsenicine (HGE) (Figure 5), belonging to the gelsedine-type alkaloid group—was described. This compound was found in honey samples collected from southern China (Guangdong Province), where numerous cases of acute poisoning and 19 deaths among 109 individuals occurred after consuming contaminated honey. Four plant-derived alkaloids were identified in the honey samples: gelsemine, gelsenicine, HGE and humantenirine. Among these, HGE accounted for more than 80% of the total toxic components. Its presence was confirmed both in the honey and in honeycombs. The average concentration of HGE in the tested samples was 17.2 µg/kg. The source of honey contamination was identified as the plant *Gelsemium elegans*. Its flowering season occurs in winter (November–January), coinciding with the timing of the poisoning incidents. This plant is frequently visited by bees, which collect its nectar and transfer toxic alkaloids into the honey. HGE proved to be a highly toxic compound: the LD_50_ for female mice was 0.125 mg/kg, and for male mice 0.295 mg/kg. It primarily affects the nervous system by enhancing the binding of GABA (gamma-aminobutyric acid) to its receptor, leading to strong inhibition of neural transmission, which may result in respiratory paralysis and death. HGE has good oral bioavailability, rapidly enters the bloodstream and brain, and is metabolized in the liver. In mouse studies, among the drugs tested, flumazenil—a GABA receptor antagonist—was the most effective in increasing survival after HGE poisoning [81].

In the study presented by Ma et al., the levels of three toxic alkaloids derived from the *G. elegans* plant—gelsemine, koumine, and humantenmine—were determined in honey samples. Thirty honey samples were collected from southern China, specifically from the provinces of Hunan, Guizhou, Yunnan, and Fujian, between January and May 2022. None of the analyzed samples contained detectable levels of the studied alkaloids. The authors suggest that this may have been due to sampling too early—before the full blooming period of *G. elegans*—or because the concentrations of these compounds were too low to exceed the method’s detection limit [82].

There is very limited researchdirectly linking gelsedine-type alkaloids with honey; however, existing information indicates that this group of compounds is highly toxic. Therefore, it should be assumed that the consumption of foods containing these substances may pose a risk to human health.

### 3.6. Tropane Alkaloids

Tropane alkaloids (TAs) are natural chemical compounds classified as secondary metabolites, produced primarily by plants of the *Solanaceae* family, but also by other families such as *Brassicaceae*, *Erythroxylaceae*, *Euphorbiaceae*, and *Convolvulaceae*. They are characterized by the presence of a tropane ring in their chemical structure, which consists of two fused rings: a pyrrolidine and a piperidine ring, connected by a shared nitrogen atom and two carbon atoms. Most tropane alkaloids are esters of organic acids and hydroxytropanols [83].

**Figure 6:**
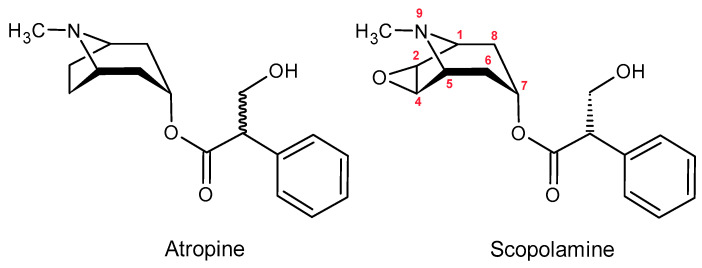
Chemical Structures of Atropine and Scopolamine.

Tropane alkaloids naturally occur in various parts of plants (seeds, fruits, flowers, leaves, stems), particularly in species such as *Datura stramonium* (jimsonweed), *Hyoscyamus*, and *Atropa* [84,85]. The most well-known tropane alkaloids in the context of food safety are atropine and scopolamine (Figure 6) [84]. These compounds are toxic and can cause severe poisoning, as they act as anticholinergic agents by inhibiting the binding of acetylcholine to muscarinic receptors. Symptoms of poisoning include tachycardia, muscle spasms, pupil dilation (mydriasis), delirium, and, in extreme cases, death [83]. TAs exhibit anticholinergic activity by inhibiting the action of acetylcholine in the central and peripheral nervous systems, primarily through blocking muscarinic receptors and, to a lesser extent, nicotinic receptors. The lethal dose of atropine is approximately 10 mg, whereas for scopolamine it is significantly lower, ranging from 2 to 4 mg [86].

There are few studies on the content of tropane alkaloids (TAs) in honey. In samples from Spain, atropine (up to 3.7 ng/g) and scopolamine (up to 5.53 ng/g) were detected. The total TA content reached a maximum of 7.2 ng/g [87]. Another study on Spanish honeys reported scopolamine at a level of 27 µg/kg [88]. TAs are not always present in honey; in a study of Polish honeys, scopolamine and atropine were not detected [89]. A study conducted by Casado et al. on 7 honey samples showed that atropine was present in all samples, while scopolamine was detected only in rosemary honeys. The total concentration of TAs ranged from 3.7 to 18.6 µg/kg [90].

There are very few studies determining the levels of TAs in honey, and the detected concentrations are very low, indicating that compounds from this group do not pose a real risk of acute toxicity after consuming honey containing these substances. However, this issue requires further research to demonstrate that honeys derived from plants rich in TAs are completely safe for human health and do not cause adverse effects during long-term exposure.

### 3.7. Tutin

**Figure 7:**
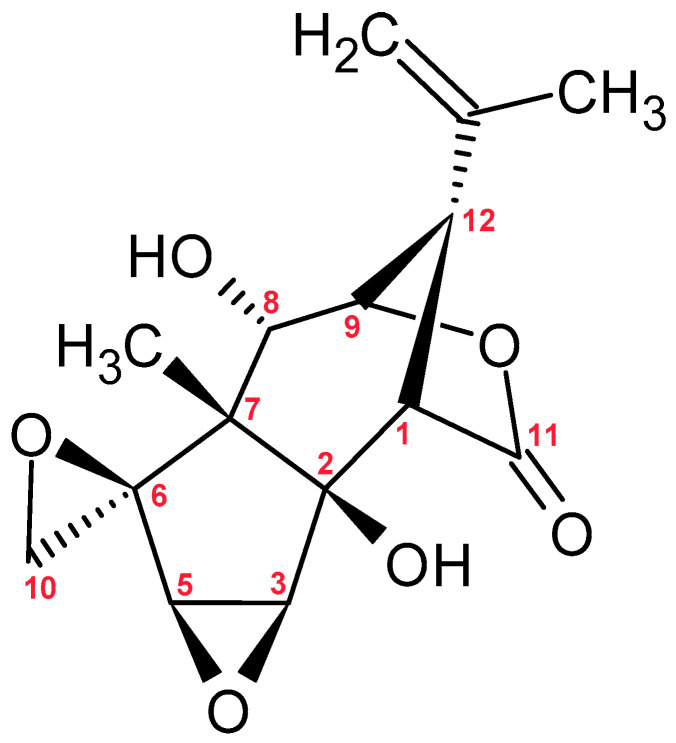
Chemical Structure of Tutin.

Tutin (Figure 7) is a picrotoxane-type sesquiterpene with a complex bicyclic structure. It consists of five- and six-ring skeleton. The molecule contains multiple hydroxyl groups, a lactone group, and an isopropenyl group [91]. Tutin is a natural toxic compound found, among others, in plants of the *Coriaria* genus, particularly in species known as *tutu*, which are endemic to New Zealand and the Chatham Islands [92]. It is known for its potent neurotoxic effects, leading to seizures and epileptic convulsions. The toxicity of tutin is primarily due to excessive activation of calcineurin, which disrupts signaling balance in the brain and results in epileptic seizures and neuronal damage [93]. The consumption of this compound can result in death [94].

In a study conducted by Larsen et al., toxic honeys from New Zealand contaminated with the neurotoxin tutin, derived from *Coriaria* spp. plants, were analyzed. Bees collected honeydew excreted by the insect *Scolypopa australis*, which feeds on these plants. In addition to free tutin, two new glycosides were detected and characterized: a tutin monoglycoside and a tutin diglycoside. In one of the most toxic honey samples, the following concentrations were measured: tutin—3.56 µg/g, hyenanchin—19.35 µg/g, tutin monoglycoside—4.93 µg/g, and tutin diglycoside—4.88 µg/g. In other samples, concentrations varied greatly—tutin reached up to 47 µg/g, and the glycosides up to 200 µg/g. The detected glycosides may be responsible for delayed toxic effects and had not been previously considered in toxicity assessments [95].

A subsequent study analyzing honey samples originating from New Zealand showed that the toxic compounds present in honeys containing tutin, hyenanchin, tutin monoglucoside, and tutin diglucoside originate from the plant *Coriaria arborea*, rather than from insect metabolism. Bees collected honeydew excreted by *S. australis* insects feeding on this plant. The presence of all four compounds was confirmed in the analyzed toxic honeys, with especially high concentrations found primarily in the plant’s phloem sink, where tutin levels reached up to 10,885 μg/g dry weight. Concentrations in the leaves and stems were significantly lower. In the honey itself, much lower amounts were detected, for example, around 8 μg/g of tutin. Moreover, the honeydew collected from *C. arborea* leaves contained significantly higher concentrations of tutin (on average 645 μg/g, ranging from 84 to 3868 μg/g) than the honey, suggesting a dilution of the toxins by nectar from other plants. The results of the study confirm that the toxic tutin glycosides have exclusively a plant origin and are not products of insect metabolism [96].

The toxicity of tutin and its effects on human health are relatively poorly understood. There is a lack of studies linking this compound to honey, which may be due to the regional distribution of plants containing this toxin.

### 3.8. Coumarins

**Figure 8:**
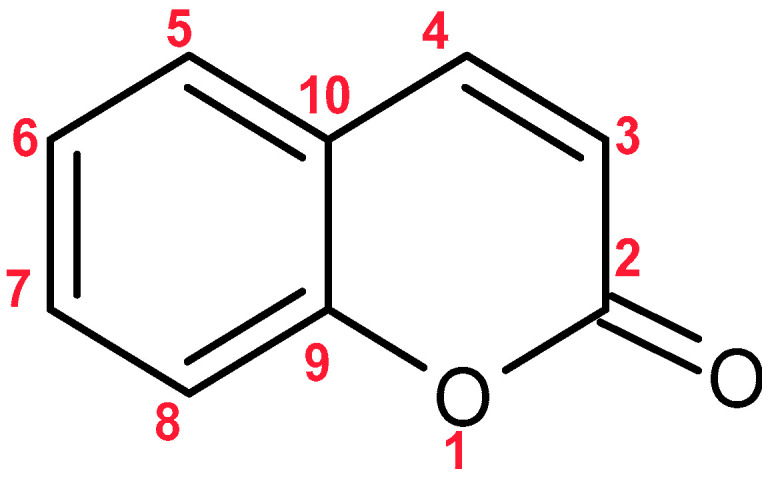
Chemical Structure of Coumarin.

Coumarins are a group of naturally occurring compounds with a benzopyrone core structure, specifically a 1,2-benzopyrone (Figure 8). The basic structure can be described as a benzene ring fused to a lactone ring (a cyclic ester), which is responsible for their chemical reactivity [97].

Dietary exposure to coumarins is high because they are present in plants. It is estimated that the intake of benzopyrones may reach approximately 1 g per day [98]. In the plant kingdom, coumarins are found in both monocotyledonous and dicotyledonous species. They are produced in significant amounts by plant families such as *Umbelliferae*, *Rutaceae*, *Compositae*, *Leguminosae*, *Oleaceae*, *Moraceae*, and *Thymelaeaceae*. These compounds are secondary metabolites of plants and are also found in certain microorganisms and animals [99]. In honey, coumarin is not a commonly encountered compound—its presence can serve as a specific botanical marker for honeys derived from certain plants. In a study of Polish yellow sweet clover honey (*Melilotus officinalis* L.), an average of 728 ng/g of coumarin was detected in two-year-old samples, with the highest concentration reaching 902 ng/g of honey. Coumarin was not detected in fresh honey, which suggests that its content increases during storage [100]. Coumarin has also been found in *Prunus mahaleb* L. honey samples and in lavender honeys [101,102]. The presence of coumarins may serve as a marker indicating the botanical origin of honey [102].

Coumarins may cause hepatotoxicity, particularly at high doses, due to the toxic metabolite coumarin 3,4-epoxide. Furanocoumarins, such as psoralen, can induce phototoxic skin reactions upon UV exposure, and in rare cases, dicoumarol may cause hemorrhages through its anticoagulant effects. Reproductive toxicity and drug interactions are also possible; however, the risk is considered low under typical consumption patterns [103,104].

Although the presence of coumarins in honey does not pose a direct health risk under typical consumption patterns, the potential for accumulation should be considered in cases of high intake of honey rich in these compounds. The literature lacks references regarding other substances from the coumarin group; therefore, it is not possible to determine whether the presence of other derivatives, such as psoralen, has any impact on the potential toxicity of honey. Currently, there are very limited data on coumarin concentrations in honey and on the corresponding toxic thresholds in humans, making it impossible to define specific intake levels or concentrations that would pose a risk (Table 1).

## 4. Other Hazardous Factors in Honey

### 4.1. 5-Hydroxymethylfurfural

**Figure 9:**
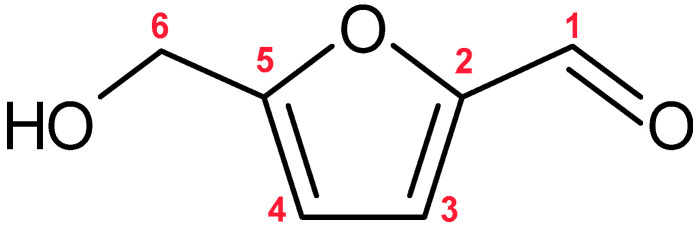
Chemical Structure of 5-Hydroxymethylfurfural.

5-Hydroxymethylfurfural (HMF) (Figure 9) is a six-carbon heterocyclic aldehyde, in which a hydroxyl group and an aldehyde group are attached to the furan ring at positions 5 and 2, respectively [105]. HMF is a potential carcinogen, as demonstrated by numerous studies [106,107]. In addition, it may trigger anaphylactic reactions and is suspected of being genotoxic [108,109]. It is present in food, where it can serve as an indicator of the intensity of thermal processing and storage duration [110]. HMF in honey is formed mainly through the dehydration of fructose and glucose, catalyzed by organic acids, low pH, increased water content, and the presence of Ca^2+^ and Mg^2+^ ions, under conditions of elevated temperature or prolonged storage. Although HMF may not be present in fresh honey, storage of the product can lead to its formation [33,111]. HMF content in honeydew honey is lower than in floral honey. This difference is not due to the botanical origin, but rather to processing practices. Floral honey is often heated to prevent crystallization and to inhibit microbial growth, which promotes HMF formation. Honeydew honey has a lower sugar content, and crystallization occurs less frequently [112]. Although heating may also be applied if necessary to inhibit yeast growth [113].

Honeys originating from different regions of Brazil, including apiaries in Caravaggio and São José do Ocoí in the state of Paraná, were analyzed for their content of HMF and furfural (FF). A total of 27 honey samples were examined, including wildflower and soybean honeys, samples from both small and large hives, honeys stored under different conditions (e.g., refrigerated, at room temperature, exposed to light), as well as commercial honeys and glucose syrups. Fresh honey samples taken directly from the hives showed acceptable levels of HMF (<36.92 mg/kg) and FF (<4.82 mg/kg). In contrast, commercial honeys exhibited significantly higher concentrations of HMF (up to 157.34 mg/kg) and FF (up to 5.28 mg/kg) [114].

In the European Union, the maximum permitted content of HMF in honey is 40 mg/kg, while for honeys originating from tropical regions, the limit is 80 mg/kg [115]. Long-term storage of honey can lead to HMF levels exceeding even 1000 mg/kg [116]. However, studies on multifloral honey from Greater Poland have shown that local products remain within the European regulatory limits [117]. This discrepancy highlights the need to monitor HMF levels in food, due to its concentration increasing over time.

HMF in honey is primarily formed through the acid-catalyzed dehydration of hexoses, in which glucose and fructose molecules are protonated by hydrogen ions derived from organic acids naturally present in honey. Protonation facilitates the opening of the sugar ring, converting it from a cyclic to a linear form. Fructose, as a ketohexose, is more reactive than glucose (an aldohexose), since its ketone structure promotes faster formation of reactive intermediates. In the linear form, the sugar undergoes enolization, transforming into an enediol intermediate. Enolization is the rate-limiting step of the reaction, and fructose enolizes more rapidly than glucose because glucose forms a more stable pyranose ring structure, which hampers ring opening. The enediol is an unstable intermediate that enables further reactions leading to dehydration. It undergoes the elimination of three water molecules, ultimately resulting in the formation of the characteristic furan ring of HMF. Although it was previously thought that HMF in honey is formed via the Maillard reaction (non-enzymatic browning, in which reducing sugars react with amino acids such as proline to form Amadori compounds and subsequently HMF), studies have shown that proline—the most abundant amino acid in honey—does not influence HMF formation. Varying proline concentrations were found to have no effect on HMF levels, thereby excluding the Maillard reaction as the dominant pathway. Instead, the acid-catalyzed dehydration process plays the key role [111].

### 4.2. Trace Metals and Metalloids

Trace metals and metalloids such as arsenic, cadmium, chromium, and lead are present in the nectar of plants that serve as an important food source for honeybees in urban environments [118]. Good beekeeping practices recommend locating hives at a safe distance from urban environments and other potential sources of contamination, including industrial areas, roads, and polluted sites [119]. Improper placement of hives can expose bees to environmental contaminants, potentially affecting both colony health and honey quality.

The presence of metals and metalloids in nectar provides a direct route of exposure for pollinators [118]. Trace metals such as cadmium, copper, and lead can alter bee behavior, particularly their responses to gustatory stimuli and food consumption [120]. Trace metals and metalloids can originate from industrial emissions, vehicle exhaust, pesticides, or natural geological processes. Bees transfer these substances to honey, reflecting the level of pollution in a given area [121,122]. The accumulation of toxic metals and metalloids poses a risk to human health, as these compounds can accumulate in the human body, causing serious health problems such as organ damage or cancer [123,124]. In the European Union, the maximum permissible level of lead in honey is 0.10 mg/kg, as set by the Codex Alimentarius and EU regulations. Cadmium is usually present at very low concentrations and a suggested limit of 0.05 mg/kg is applied, although it is not uniformly regulated in honey. For arsenic, the EU has established a general food limit of 0.1 mg/kg, which also applies to honey. Mercury is typically found at trace levels, and while the EU does not specify a strict honey standard, some national regulations allow up to 0.01–0.02 mg/kg. In addition, essential trace elements such as copper and zinc should not exceed 2 mg/kg and 5 mg/kg, respectively, in accordance with FAO recommendations [125].

A study conducted in Lithuania analyzed honey samples collected during the summer of 2020 from twelve distinct locations, selected based on their proximity to potential pollution sources such as industrial plants, landfills, major roads, and railway lines. The highest concentrations of heavy metals—including cadmium, lead, chromium, copper, and nickel—were found in honey from sites located closest to these pollution sources. Notably, the highest lead content (1.6498 mg/kg) was detected in a sample collected from a hive situated between the Vilnius-Klaipėda railway line and the Vilnius-Kaunas highway, just 0.3 km and 0.1 km away, respectively. In contrast, the lowest levels of heavy metals were observed in honey samples from background locations such as the Čepkeliai Reserve and Mizarai village, which were considered unpolluted. Statistical analysis, including Spearman’s rank correlation, confirmed a significant negative correlation between heavy metal concentrations and the distance from pollution sources, supporting the hypothesis that contamination levels decrease with increasing distance from anthropogenic activity [126].

A comprehensive analysis of 28 honey samples collected between 2022 and 2023 from 15 U.S. states revealed elevated concentrations of toxic metals and metalloids, raising potential concerns for consumer health. Lead levels reached up to 0.918 µg/g, cadmium up to 0.918 µg/g, mercury up to 8.94 µg/g, and arsenic up to 0.067 µg/g—values that in many cases exceeded the maximum residue limits established by the European Union. In total, 20 elements were quantified using benchtop X-ray fluorescence, including essential minerals such as copper, iron, and zinc. While concentrations varied widely, most non-toxic elements were present at levels below 1 µg/g, with exceptions such as sodium (mean: 406.8 µg/g), calcium (234.8 µg/g), and zinc (75.9 µg/g), which were consistently higher across samples [127]. A study conducted on six honey samples from the South Wollo Zone in Ethiopia demonstrated low concentrations of selected heavy metals, suggesting minimal environmental contamination and confirming the safety of these honeys for human consumption. Zinc levels ranged from 1.97 to 2.04 µg/g, copper from 1.92 to 2.02 µg/g, manganese from 0.83 to 1.01 µg/g, chromium from 0.25 to 0.45 µg/g, and cadmium from 0.025 to 0.031 µg/g. Notably, lead was not detected in any of the analyzed samples. These values fall well below the maximum permissible limits established by international food safety standards, indicating that honey produced in this region is of high quality and poses no toxicological risk [128]. Another study from Ethiopia found higher chromium levels of 6.66 µg/g. Contact between honey and stainless-steel surfaces, which may be prone to corrosion under certain conditions, has been suggested as a potential source of this metal in honey [129].

Trace metals and metalloids present in honey may pose a risk to human health due to their potential to accumulate in the body. However, literature data generally indicate that the levels of these compounds are safe and should not pose a health concern under normal consumption conditions.

### 4.3. Pesticides

Due to the widespread use of pesticides to increase crop yields, these substances are also detected in bee products [130,131]. These compounds may have adverse effects on bees [132,133]. Pesticides have a harmful impact on bees both through direct poisoning and chronic exposure of entire colonies to contaminated food sources. Poisoning often results from spraying during the flowering of rapeseed, and toxic cocktails of substances—especially chlorpyrifos, pyrethroids, and neonicotinoids—can lead to bee mortality and weakened immune systems [134]. Honey contaminated with pesticides, although it may look and taste normal, poses a real threat to consumer health. This harmfulness results not only from individual substances but also from their synergistic effects and accumulation in the body. Pesticides present in honey can exert toxic effects on the human body, causing DNA damage, hormonal imbalances, and weakening of the immune system. Long-term consumption of such honey may increase the risk of developing cancers such as breast cancer, lung cancer, or leukemia [135].

Honey can be a useful bioindicator of environmental pesticide contamination because pesticide levels in honey reflect those in the environment [136].

The maximum residue levels (MRLs) of pesticides in honey are regulated by the European Union under Regulation (EC) No 396/2005 on pesticide residues in food and feed, which sets a general limit of 0.05 mg/kg for most compounds, while allowing higher or lower thresholds for selected substances [137].

In samples of varietal honey from southeastern Poland, residues of eleven chemical substances classified as pesticides were detected. The most frequently identified insecticides were thiacloprid (90% of samples; max. 0.337 mg/kg) and acetamiprid (86.6%; max. 0.061 mg/kg). Among the fungicides, carbendazim was the most prevalent (60%) [138]. In honey samples from apiaries affected by bee poisoning incidents in eastern China, residues of six chemical substances classified as pesticides were detected. The most frequently identified compounds were the fungicides carbendazim (99.0%; max. 5.5 µg/kg) and semiamitraz (93.8%; max. 9.0 µg/kg), as well as the insecticide acetamiprid (49.0%; max. 7.9 µg/kg). In 95.9% of the samples, the co-occurrence of at least two pesticides was observed [139].

As part of a three-year monitoring program conducted between 2020 and 2022, 221 honey samples from apiaries located in the Lombardy and Emilia-Romagna regions of Italy, representing both conventional and organic farms, were analyzed. The study revealed the presence of polar pesticide residues, including glyphosate, aluminium fosetyl, and its metabolite—phosphonic acid. Glyphosate was the most frequently detected compound, found in approximately 28% of the samples; in two cases, its concentration exceeded the permissible limit of 0.05 mg/kg, reaching up to 0.31 mg/kg. Notably, traces of this herbicide were also identified in honey from organic apiaries, suggesting the existence of environmental contamination sources independent of production practices [140].

Pesticides are a widely prevalent group of compounds found in honey. Their accumulation in the human body can be toxic due to the harmful nature of these substances.

### 4.4. Clostridium botulinum

*Clostridium botulinum* spores are ubiquitous worldwide. These are Gram-positive bacteria that produce protein, botulinum neurotoxins (BoNT) resistant to gastric juice and proteolytic enzymes in the digestive system. The toxins secreted by these bacteria are exceptionally heat-stable [141]. Neurotoxins are classified into 9 toxinotypes and 41 subtypes [142,143]. BoNT causes muscle paralysis. Due to the route of exposure through food, this condition is classified as foodborne botulism [144]. Infants are a particularly vulnerable group to botulism due to the immaturity of their gut microbiome and the ease with which *C. botulinum* can colonize their intestines [142]. Infant botulism occurs sporadically worldwide [144].

Microorganisms present in honey must be resistant to high sugar content and antimicrobial compounds. Honey can become contaminated through the transfer of microorganisms along with pollen; they may also originate from the bee’s digestive tract. Contamination can also result from dirt, dust, and non-sterile conditions during honey processing [145].

A study conducted on honey from various regions of Poland revealed the presence of *C. botulinum* in 5 out of 240 tested specimens. Additionally, bacterial strains with phenotypic similarity to *C. botulinum* were identified; however, they did not show the ability to produce BoNT [146]. In different study, 102 honey samples from small Polish apiaries were analyzed, detecting *C. botulinum* spores in 22 samples. The average number of spores was 190 per gram of honey, with a maximum value of 1800 in multifloral honey [147]. Another study on honey originating from Kazakhstan detected the presence of *C. botulinum* in 1 out of 197 samples. In addition, *Clostridium perfringens* was identified in 18 of the tested specimens [145].

The presence of C. botulinum in honey does not pose a health risk to healthy adults. However, honey must not be given to infants due to the risk of botulism. Individuals with weakened immune systems or serious illnesses should also avoid raw honey.

### 4.5. Allergens

Honey, due to its diverse composition, may cause allergies. Allergens may be of plant origin, come from insects, or be contaminants. If hypersensitivity occurs, it may result in anaphylaxis, which poses a threat to life and health [148]. Honey allergies can manifest as mild itching in the oral cavity to severe systemic reactions, including tongue swelling. A study conducted on the sera of patients with honey allergy showed that IgE antibodies bound to proteins derived from the secretions of bee salivary and pharyngeal glands as well as plant pollen [149]. A case of an allergic reaction in a child after honey consumption has been reported. It was most likely triggered by pollen from plants of the *Asteraceae* family [150]. Honey allergies are rare, with only a few cases reported in the literature [151,152,153].

Trace amounts of gluten and milk proteins have also been detected in honey, most likely originating from cross-contamination during production or packaging, including supplementary bee feeding. The gluten levels were low enough for the honey to be considered safe for individuals with gluten sensitivity, as all samples fell below the EU threshold of 20 ppm. Regarding milk allergens, although the detected levels of Bos d 5 and Bos d 11 were low and below the ED01 threshold (0.2 mg per serving), which is considered safe for the majority of milk-allergic individuals, the presence of these proteins may still be of concern for highly sensitive individuals. Therefore, routine allergen testing may be advisable, especially in facilities handling milk-containing products [154].

### 4.6. Miscellaneous (PAHs, PCBs, PFAS, Mycotoxins, Veterinary Drug Residues)

In addition to the compounds discussed earlier, honey contains a wide range of other harmful substances. Environmental contaminants include, among others, polyaromatic hydrocarbons (PAHs) [155,156], chlorinated organic biphenyls (PCBs) [157,158], and perfluorinated alkylated substances (PFAS) [159,160]. Although reports on these components in the literature are scarce, they are found in very small amounts due to their widespread presence in the environment. Furthermore, mycotoxins produced by molds and fungi have also been detected [161,162].

Attention should also be paid to the presence of pharmaceutical and veterinary drug residues, such as antibiotics used to treat bee diseases [39,163,164]. Another important group of contaminants in honey are acaricides, synthetic substances applied in beekeeping to control *Varroa destructor*, a parasitic mite that poses one of the most serious threats to honeybee colonies. Due to the widespread problem of resistance, treatments are often applied at higher-than-recommended doses, which increases the likelihood of residues being present in bee products. Analytical surveys have confirmed their occurrence in honeys of various botanical origins. For example, residues of τ-fluvalinate were detected in multifloral and rosemary honeys at concentrations ranging from below the limit of quantification up to 23 µg/kg, while in heather honey the levels did not exceed the method’s quantification limit. These concentrations were below the MRLs established by the European Union (50 µg/kg). Although the amounts detected are typically low, the persistence of certain acaricides, such as τ-fluvalinate, is concerning, since its residues may remain stable in honey for many months under storage conditions [165]. Recent monitoring in Spanish apiaries also revealed that amitraz and coumaphos are among the most prevalent residues in hive products. Amitraz, detected through its metabolites (DMF and DMPF), reached concentrations up to 396 µg/kg in honey, exceeding the EU MRL of 200 µg/kg, although levels decreased significantly after several months of storage. Coumaphos was present less frequently, typically below 15 µg/kg in honey, but its strong lipophilicity favors accumulation in wax, from which it can migrate into brood and honey over time. Notably, residues of banned compounds such as acrinathrin and chlorfenvinphos have occasionally been detected in honey at concentrations up to 24.5 µg/kg, pointing to illegal use or contamination from recycled wax foundations [165]. Veterinary drugs may enter honey as a result of improper use or failure to observe the required withdrawal periods. Even trace amounts raise justified concerns due to the risk of allergic reactions, disturbances of the gut microbiota, and the development of bacterial resistance to antibiotics, which is a serious public health issue.

Although the risk of acute toxicity is negligible, the accumulation of these substances and their potential interactions may contribute to chronic toxicity, which still requires further investigation (Table 2).

## 5. Conclusions

Honey is globally recognized as a valuable dietary product with a wide spectrum of health-promoting properties, and the analysis presented in this work highlights that its consumption is not free from toxicological concerns. Natural toxins of plant origin (including pyrrolizidine alkaloids, grayanotoxins, triptolide, celastrol, gelsedine-type alkaloids, tropane alkaloids, and tutin) may compromise the safety of honey by exerting hepatotoxic, neurotoxic, and cardiotoxic effects, with severe intoxications occasionally resulting in fatalities. In parallel, environmental pollutants and anthropogenic contaminants (heavy metals, pesticides, HMF, antibiotics, mycotoxins, allergens) represent additional risk factors that can accumulate in the human body, leading to chronic toxicity, DNA damage, immunosuppression, or allergic responses.

Therefore, the toxicological profile of honey must be considered alongside its beneficial health effects. Increased awareness, stricter quality control, and advanced analytical monitoring are required to ensure consumer safety, particularly in the context of global trade and growing demand for natural products. This risk strongly depends on the local vegetation, beekeeping practices, environmental pollution levels, and honey storage conditions. Although the likelihood of acute toxicity is generally low, the potential for chronic health effects resulting from long-term consumption of contaminated honey should not be underestimated. Many studies focus on acute toxicity, often overlooking the impact of chronic exposure to phytotoxins. Moreover, limited public awareness and the widespread perception of honey as a “superfood” may contribute to downplaying the existing risks.

Raising consumer awareness and implementing effective monitoring and quality control methods for honey products available on the market is essential. Legal regulations could help eliminate toxic batches from commercial distribution. Testing commercially available honey is a good strategy to reduce the risk of poisoning. Avoiding the consumption of honey from unknown sources also represents a promising form of prevention against acute intoxication.

Another growing concern is the potential presence of unidentified phytotoxins in honey that have not yet been described in the scientific literature. This area presents an important research gap that should be addressed in future studies.

## Figures and Tables

**Table 1:** Summary of plant-based toxic compounds detected in honey, their estimated probability of occurrence, and the associated harmful health effects.

Compound	Probability of Ingestion	Details	Harmful Effects
pyrrolizidine alkaloids	medium-high	-	cancerogenic, hepatotoxicity
grayanotoxins	low	regionally high	cardiotoxicity, genotoxicity
triptolide	low	regionally high	organ toxicity, death
celastrol	low	regionally high	hepatotoxicity, neurotoxicity
gelsedine-type alkaloids	low	regionally high	neurotoxicity
tutin	low	regionally high	neurotoxicity
tropane alkaloids	medium	-	N/A ^1^
coumarins	high	-	probably hepatotoxicity

^1^ Unknown for the doses found in honey.

**Table 2:** Summary of other toxic compounds detected in honey, their estimated probability of occurrence, and the associated harmful health effects.

Compound	Probability of Ingestion	Details	Harmful Effects
5-hydroxymethylfurfural (HMF)	high	increases with storage time	cancerogenic
trace metals	medium	-	chronic toxicity
pesticides	high	-	chronic toxicity
*C. botulinum* spores	medium	-	infant botulism
allergens	high	extremely rare cases	allergies (extremely rare)
PAHs, PCBs, PFAS, mycotoxins, veterinary drug residues	high	probably cumulative	N/A ^1^ (probably chronic toxicity)

^1^ Unknown for the doses found in honey.
